# Benign Tumors and Cysts1Read at the State meeting of the Michigan Veterinary Medical Association, at Lansing, Michigan, February 5, 1901.

**Published:** 1901-06

**Authors:** H. F. Palmer

**Affiliations:** Detroit, Mich.


### BENIGN TUMORS AND CYSTS.[1]

By H. F. Palmer, B.S., D.V.S.,
DETROIT, MICH.

A tumor is any circumscribed enlargement or swelling or any disease in which portions of the body depart from their normal form by an unnatural increase in size. It is also more restricted and is applied only to those new formations of obscure origin which, after appearance, remain either as permanent or progressive productions.

Tumors are of two kinds—benign and malignant. A benign or non-malignant tumor is one which, if allowed to remain, is capable of doing injury only by its bulk and pressure, or which, if removed, exhibits no tendency to return.

A malignant tumor is one which proves destructive to the tissue in which it is located, which tends to contaminate adjoining glands or to be disseminated through the bloodvessels, including changes unfavorable to the proper nutrition of the body.

After removal they manifest a tendency to recur either at the original seat of the disease or in another part of the body, and are capable eventually of destroying life. The number of malignant as compared to the number of benign tumors is fortunately small.

The benign tumors do not materially differ from the nature of the tissue in which they fix their habitation. A lipoma, which is a neoplasm of fat, is in most respects identical with the surrounding adipose tissue, and a fibroma is only a localized hypertrophy of the connective tissue in the midst of which it is lodged. Malignant tumors, on the contrary, embody in their structure histological conditions altogether unlike the perfected tissue in which they grow.

The following considerations will help us to differentiate the two kinds of tumors: Malignant tumors are solitary and recur after extirpation. Benign tumors have not the glandular infection and do not infiltrate into other tissues. Benign tumors develop slowly and do not adhere to skin and adjoining tissues. Both kinds of tumors usually pursue a painless growth for a time, and the benign seldom cause pain unless from their bulk interfering with natural functions. Benign tumors have a feeble blood-supply, and they seldom contain any juice.

[1] Read at the State meeting of the Michigan Veterinary Medical Association, at Lansing, Michigan, February 5, 1901.

Several theories have been advanced accounting for the origin of tumors. The congenital, the nervous influence, spontaneous, and inflammatory theories all have their advocates. Whatever the growth may be there are three conditions necessary for the development of a morbid growth—structural peculiarity, a specific irritant, and inflammation.

Benign tumors are classified as follows :

Fibroma of fibrous material.
Epithelioma of skin.
Osteoma of bone.
Enchondroma of cartilage.
Neuroma of nerves.
Lipoma of adipose tissue.
Adenoma of secreting glands.
Myxoma of mucous tissue.
Angioma of bloodvessels.
Myoma of muscle.
Lymphoma of lymphatic tissue.

Fibroma or fibrous tumor is usually found in parts containing much fibrous tissue. It is a hard, rounded, painless tumor. Surface of tumor smooth or divided into lobes, generally movable, and contained in a wall of areolar tissue. They vary in size from that of a grain of shot to a goose-egg, or even larger. They are made up of fibres of yellow elastic and white fibrous tissue, which run in various and apparently confused directions. The compactness of these layers of fibre gives the tumor a hard or soft nature. They have few bloodvessels and are usually lodged in a sac and receive their nourishment from its walls. The favorite seat of these tumors is on the inferior surface of the abdomen, and also in the uterus, especially of the bitch.

*Diagnosis.* Slowness of growth, hardness of structure, and regularity of surface, absence of pain except when the tumor presses on the adjacent nerves, no tendency to become adherent to the integument, no enlarged veins over the surface.

*Treatment.* When they mature remove with knife or écraseur. They can often be removed by making an incision in the skin and pressing them out by hand and dressing the sac with some astringent, as copper sulphate.

Nasal polypi are a species of fibrous tumors attached by a narrow pedicle. They are of soft nature, bleeding when injured, growing downward and fill the nasal cavity. The diagnostic symptoms of this are : discharge from the nostril often tinged with blood, sniffling sounds in breathing, and frequently sneezes. They may grow backward and fall into the isthmus of fauces. Tumor may not be seen.

*Treatment.* Remove by some means and wash with astringent lotion. Fibrous tumors may be due to imprisonment of pus in the deep-seated intermuscular structures. These can be removed by the knife or by caustics, the former method being preferable in large tumors.

*Epithelioma* includes all warts, corns, horns, and papilloma. Warts consist of a thickening of the epidermis produced by accumulation of its scales with hypertrophy of the papillæ of the true skin. They are found mostly on young animals, their favorite place being the under surface of the abdomen, the genitals, lips, and eyelids.

They may be removed either by excision, torsion, or by caustics. Warts of large nature can be easily removed by putting a tight rubber band around the base, thus shutting off the blood-supply and sloughing off the wart. To remove a wart by caustics first wash it thoroughly, and then apply any of the strong mineral acids, taking care not to let any of the acid come in contact with the surrounding skin. Usually one application suffices.

A soft papillomata or myxoma consists of products of the mucous membrane, and is located on any of the mucous membranes, but especially on those of the mouth and vagina. These can be extirpated in the same manner as the soft papillomata, provided they are situated in an accessible place.

*Adenoma* is an epithelioma, and is a new formation or development of gland tissue. These tumors vary as the structure of the glands vary. Some are racemose and some tubular, depending on the epithelium which lines the gland. These tumors are benign in their nature and exhibit no tendency to return when thoroughly removed. Although often mistaken for carcinoma, they can be differentiated by noting that in adenoma the cells lining the cavities of the glands rest on a well-defined basement membrane, which is not the case in carcinoma. As the cause of adenoma is some local irritant, the remedy that suggests itself is an operation to remove that irritant.

*Lipoma*, or fatty tumor, consists of a quantity of normal fat cells closely packed together. They are not very well supplied with bloodvessels or nerves, and hence this makes their removal easier.

They can be removed by excision, and the sac must be broken down and destroyed by the actual cautery or a caustic wash.

*Neuromatous* tumors are not as frequent in our patients as in the human family. Only one set of nerves gives us much trouble from such attacks. This is the plantar after its division in foot-lameness. These tumors are of a solid, firm consistency, composed of a fibrous stroma, with numerous groups of cells scattered throughout them. They are found as round or oval bodies with their long diameter along the course of the nerves, movable in transverse but not in long direction. Being a part of the nerve, they cause great pain, and should be excised at once.

*Enchondroma,* or cartilaginous tumors, consist of any or all of the three kinds of cartilage—hyaline, fibrous, and mucoid—the favorite seat of these tumors being in the region of the sternum or upon the ribs. Their peculiar location is due perhaps to their cause, which is often from an external injury. There are two forms, one being round or oval with well-defined limits, the other having no well-defined border, but resembles an infiltration into the surrounding tissue. These latter may arise from the development and growth of cartilage in an inflammatory exudate.

The diagnosis of enchondromatous tumors is not generally a matter of difficulty. Their hard, slightly compressible feeling, their knobbed or irregular surface, their painless progress, and usually their connection to cartilage or bone. The treatment consists in their removal as early as possible by excision, as their location, especially on the sternum, may cause great inconvenience to the animal's movements.

*Osteoma.* These tumors, commonly described as exostoses, are masses of bone or outgrowth from different portions of the skeleton. In structure and chemical composition they agree with either the compact or spongy tissue of normal bone. In form they are not uniform, being sometimes lobulated, sometimes spherical, and at other times spinous or spiculated. They are of slow growth, and vary much in size. Except when the tumor attains considerable magnitude, little inconvenience is experienced by the animal, and when pain accompanies them it is due to their pressure on adjacent nerves, or by interference with the movement of tendons. Their extreme hardness and firm connection with bone are their chief characteristics. Being of slow growth, benign in their character, rarely attaining to any great magnitude, and having a tendency to become fixed, it seldom becomes necessary to remove them. If they do attain to such a size that their removal is necessary, they can be uncovered and detached with a saw. Even when they are pedunculated and a portion of their stump is left the base shows no disposition to grow.

A *myoma* is a tumor consisting exclusively of muscular tissue. They are exceedingly rare, but may occur in two forms. One is allied in its histological structure to striated muscle, but having very much smaller fibre cells; the other is simply a hypertrophy of muscular tissue. The former are usually found in some portion of the genito-urinary tract. The latter are associated with a preponderance of connective tissue resembling a fibroma, and hence often called fibromyoma. These occur in the uterus, vagina, bladder, testicle, prostate, scrotum, œsophagus, stomach, and intestines. The tumor is firm in consistence, being spherical or pyriform, and has a white or flesh-colored appearance when laid open. The growth of these tumors is often arrested, and in some cases diminished by the use of ergot. This acts by inducing muscular contraction, and hence lessens blood-supply. If the growth is of such a size that it hinders the animal or jeopardizes its life, they may be extirpated either through natural channels or through an abdominal incision.

*Lymphoma* may be divided into two classes—the soft and hard. In the soft all the cells of the lymph gland are greatly increased in size and number, while in the hard kind there is a preponderance of connective tissue and a diminished number of lymphoid cells.

The diagnostic symptoms of lymphoma are that they usually occur in thrifty young animals, and affect a whole chain of glands of a limited area of one side of the body. They have no tendency to suppuration or caseous change, and do not excite inflammation in surrounding tissue. A lymphoma under any circumstances gives just cause for apprehension, and, if constitutional contamination occurs, the case is as hopeless as carcinoma.

*Treatment.* Iodine and arsenic both internally and externally and iodoform dressing. Their removal with the knife is usually unsatisfactory.

*Angioma* is a tumor composed of bloodvessels supported by connective tissue, and is known under various names, as erectile tissue, aneurism by anastomosis, etc. These are helped and often removed by application of the actual cautery or by enucleation.

All cysts may be cysts divided into two general classes—retention cysts and neoplasmic cysts. The former consist in a distention and hypertrophy of a duct or secreting gland, the contents of which are the normal secretion more or less altered by retention.

The latter are new formations which are the result of enlargement of the primitive cell or areolar interspace.

Retention cysts occur on the skin and subcutaneous cellular tissue. They are round and smooth in outline and free from pain. They are composed of sebaceous secretion, epithelial scales, oil globules, granular matter, and crystals of cholesterin.

To this class belong mucous cysts, sebaceous cysts, salivary cysts, distention of bursa, effusions into sheaths of tendons, and muscles.

*Mucous cysts* may appear anywhere on a mucous surface. They are tense, globular swellings, occasioning little or no pain, and are filled with viscid, ropy mucus and epithelial débris.

*Sebaceous cysts* are quite common in the human family, especially among the females. Nearly all have recently seen specimens of these and the manner of their removal, so they need no further description here.

*Salivary cysts* are met with in connection with the ducts of the salivary glands. When these appear under the tongue on either side of the frænum lingui they are then called ranula.

*Distention of bursa* is quite common about the posterior part of the fetlock-joint, and is known as wind-galls. They are soft, symmetrical cysts of varying dimensions, painless, and, except for their unsightly appearance, cannot be called an unsoundness.

Distention of bursa may be seen better developed and causing more inconvenience in the region of the hock. These cause lameness by their mechanical obstruction to the free movement of the joint. Among these may be noted thoroughpin, which in many cases is simply a distention of the bursa.

All retention cysts are treated by producing absorption by means of stimulating applications. If this fails tap the cyst, remove its contents, and scrape the internal membrane or inject a stimulating fluid into the sac after the contents are removed; or, if in a favorable location, they may be dissected out. The latter method is probably the preferable one.

A neoplasmic cyst is filled with a fluid secreted by its lining membrane. The fluid may be thin or serous, thick or viscid. These cysts may be simple or compound. The compound often enclose teeth, hair, etc. When enclosing teeth they are called dentigerous cysts.

The simple cysts may include capped hock, knee, elbow, and fetlock, which may all be considered under one head. They all are usually the result of some external injury. At first there is a mere fusion of serum underneath the skin. This terminates in a serous abscess named from its location.

Treatment may consist in the application of the following:

℞.—Hydrarg. biniodid. . . . . . . . ʒij.

Water . . . . . . . . . . ℥xij.

Pot. iodide . . . q. s. to dissolve the mercury.

Use until soreness occurs, then withhold and apply later. If this fails, may aspirate, use seton, or else perform a radical operation.

*Bronchocele*, Derbyshire neck, or goitre. These are cystic enlargements of the thyroid gland, the origin of which is unknown. They are said to originate from the drinking-water taken into the system. It is a soft fluctuating swelling of the thyroid gland, occupying one or both sides of the larynx. Although unsightly, they never interfere with the general usefulness of the animal.

*Treatment:*

℞.—Iodine . . . . . . . . . . ʒj.

Pot. iodide . . . . . . . . . ʒj.

Lard . . . . . . . . . . ʒviij.

Apply once a day until soreness is produced. If very obstinate, give one ounce of potassium iodide three times a day for eight days.

Parasitic cysts are a class of the compound neoplasmic cysts. These are cysts containing larvæ of some parasite.

Cysts containing teeth are found in the testicles and other parts of the body, but the favorite locality of these is within the antrum even as high up as the base of the ear. The development of these dental tumors is due to some malformation during fœtal life. The teeth continue to grow in their unnatural location, and so form immense tumors.

Simple neoplasmic cysts can be treated the same as retention cysts by applying stimulating applications or drawing off the contents and injecting stimulating medicines. The compound neoplasmic cysts have but one method of treatment and that is excision. They must be thoroughly and effectually dissected away.

---

The American Public Health Association will hold its convention this year in connection with the Pan-American Exposition at Buffalo, N. Y., September 17th to 20th.

